# Isolation of *Salmonella* Mutants Resistant to the Inhibitory Effect of Salicylidene acylhydrazides on Flagella-Mediated Motility

**DOI:** 10.1371/journal.pone.0052179

**Published:** 2013-01-02

**Authors:** Isabel Martinez-Argudo, Andreas K. J. Veenendaal, Xia Liu, A. Dorothea Roehrich, Maria C. Ronessen, Giulia Franzoni, Katerine N. van Rietschoten, Yusuke V. Morimoto, Yumiko Saijo-Hamano, Matthew B. Avison, David J. Studholme, Keiichi Namba, Tohru Minamino, Ariel J. Blocker

**Affiliations:** 1 Schools of Cellular & Molecular Medicine and Biochemistry, University of Bristol, Bristol, United Kingdom; 2 Sir William Dunn School of Pathology, University of Oxford, Oxford, United Kingdom; 3 Graduate School of Frontier Biosciences, University of Osaka, Osaka, Japan; 4 Biosciences, College of Life and Environmental Sciences, University of Exeter, Exeter, United Kingdom; 5 Precursory Research for Embryonic Science and Technology, Japan Science and Technology Agency, Kawaguchi, Saitama, Japan; University of Osnabrueck, Germany

## Abstract

Salicylidene acylhydrazides identified as inhibitors of virulence-mediating type III secretion systems (T3SSs) potentially target their inner membrane export apparatus. They also lead to inhibition of flagellar T3SS-mediated swimming motility in *Salmonella enterica* serovar. Typhimurium. We show that INP0404 and INP0405 act by reducing the number of flagella/cell. These molecules still inhibit motility of a *Salmonella* Δ*fliH-fliI-fliJ/flhB*_(P28T)_ strain, which lacks three soluble components of the flagellar T3S apparatus, suggesting that they are not the target of this drug family. We implemented a genetic screen to search for the inhibitors' molecular target(s) using motility assays in the Δ*fliH-fliI/flhB*_(P28T)_ background. Both mutants identified were more motile than the background strain in the absence of the drugs, although HM18 was considerably more so. HM18 was more motile than its parent strain in the presence of both drugs while DI15 was only insensitive to INP0405. HM18 was hypermotile due to hyperflagellation, whereas DI15 was not hyperflagellated. HM18 was also resistant to a growth defect induced by high concentrations of the drugs. Whole-genome resequencing of HM18 indicated two alterations within protein coding regions, including one within *atpB*, which encodes the inner membrane a-subunit of the F_O_F_1_-ATP synthase. Reverse genetics indicated that the alteration in *atpB* was responsible for all of HM18's phenotypes. Genome sequencing of DI15 uncovered a single A562P mutation within a gene encoding the flagellar inner membrane protein FlhA, the direct role of which in mediating drug insensitivity could not be confirmed. We discuss the implications of these findings in terms of T3SS export apparatus function and drug target identification.

## Introduction

To battle the increasing antibiotic resistance of pathogenic bacteria, it is crucial to develop new antimicrobial agents. Strategies relying on existing targets and drugs, which are often derivatives of compounds that microorganisms use to combat each other and which directly affect bacterial viability, all face the same problem. Resistance to the drug(s) has often already emerged in the wild and quickly spreads under the huge selective pressure [1]. Structurally novel drugs, that specifically target virulence properties without killing bacteria and are hence unlikely to have been previously used in nature, might decrease the chance of bacterial resistance emerging as quickly [2]. Such compounds might also have the advantage of sparing commensals, further reducing the likelihood of resistance emergence and also decreasing the risk of side effects associated with depleting the normal flora. However, a potential disadvantage of pathogenic mechanisms as therapeutic targets is that many are microbe-specific, necessitating more rapid and costly pathogen identification than is available in clinical practice at present.

Type III secretion systems (T3SSs) are encoded by approximately 25 genes, which share homology with those encoding bacterial flagellar basal bodies [3]. Upon direct physical contact with host cells, T3SSs are induced to secrete and translocate protein effectors of virulence, from the bacterial cytoplasm into the host cell cytoplasm. They are prime target candidates for “antivirulence” compounds because they are so broadly distributed across Gram-negative bacterial pathogens of plants, animals and humans, where they are often essential to virulence. However, they are also found in a number of commensals albeit often with unknown functions [4]. In recent years, whole-cell based high-throughput screens have been performed to identify inhibitors of T3SSs [5], [6], [7], [8], [9], [10]. These screens have identified several classes of synthetic compounds (salicylidene acylhydrazides, salicylanilides, sulfonylaminobenzanilides, benzimidazoles and a thiazolidinone) and three natural products (glycolipid caminosides, guadinomines and the linear polyketide antibiotic aurodox at concentrations not affecting bacterial viability) as active for inhibition of T3SSs in a range of Gram negative bacterial pathogens, including *Yersinia*, *Chlamydia*, and *Salmonella* [5], [6], [7], [9], [10], [11], [12], [13], [14], [15], [16], [17], [18], [19], [20], [21].

Some salicylanilides and sulfonylaminobenzanilides likely inhibit T3SS gene transcription in EPEC [5] or *Yersinia* and seem very species-specific [6], [11]. A few benzimidazoles have been shown to inhibit transcription of multiple adaptational response family transcription factors (including LcrF of *Yersinia* and ExsA of *Pseudomonas*) and may therefore have a broader range [9], [10], [18]. A 2-imino-5-arylidene thiazolidinone compound inhibited virulence-associated T3SSs, but not flagellar biogenesis ones, and also affected type II secretion systems. This suggests that its target is an outer membrane component conserved between these two types of secretion system but not found in flagellar biogenesis ones [7]. The other T3SS inhibitors, including all identified natural products [12], [13], [19], [21] have unknown targets [8], [19], [20].

The salicylidene acylhydrazides were found to inhibit expression and secretion of T3SS-related proteins and to affect host cell infection by *Yersinia*, *Chlamydia*, and *Salmonella* [14], [15], [16], [17], [22], [23]. These T3SS inhibitors were also found to reduce the motility of *Yersinia* and *Salmonella* [11], [14] and to decrease flagellin expression and surface localization in *Salmonella* [14]. Taken together, these findings indicate that the inhibitors act on a conserved target, since they interfere with both the virulence T3SSs and the genetically, structurally and morphologically related bacterial flagellar export systems that mediate motility. We showed that these compounds have a detrimental effect on T3SS needle assembly, as demonstrated by increased numbers of T3SSs with shorter or without needles [24]. Therefore, the compounds generate a phenocopy of T3SS export apparatus mutants, but with incomplete penetrance. Given the known assembly checkpoints for T3SSs and flagella [25], this would be sufficient to almost completely block the later secretion of effector proteins.

Further development of any of these prototype T3SS inhibitors into novel drugs requires that their precise target(s) are identified to allow directed small molecule improvement. Efforts have so far focused on the initially described salicylidene acylhydrazides. These compounds were shown to alter T3SS gene expression in *E. coli* O157 [26] and their effect on the *Chlamydia trachomatis* and *Salmonella* SPI1 T3SS can be reversed by iron [27], [28], although regulation of iron metabolism genes is unaffected by inhibitor addition in *E. coli*. Recently, an affinity chromatography approach allowed identification of three *E. coli* proteins that interact directly with salicylidene acylhydrazides compounds: WrbA, an inner membrane NADPH-dependent FMN reductase which is a peripheral component of the electron transport chain; Tpx, a cytoplasmic/periplasmic thiol peroxidase involved in response to oxidative stress and FolX, an dihydroneopterin-tri-P-epimerase, the biological role of which is unclear [29]. By transcriptomic analysis, deletion of these genes was shown to affect flagellar and virulence T3SS gene regulation, suggesting the drugs work by indirect and synergistic effects on T3SS regulation.

We took a different approach, seeking to establish a system to allow easy genetic screening for mutants resistant to the action(s) of salicylidene acylhydrazides on T3SS function. We used the flagellar biogenesis system in *Salmonella* because it is the best-characterized T3SS genetically, functionally and structurally (reviewed in [25]) and because motility induced by assembled flagella leads to an economical and convenient visual screening method.

For flagellum assembly, component proteins are transported to the distal end of the growing structure by the flagellar type III protein export apparatus. This consists of three soluble proteins FliI, FliH, FliJ, and six inner membrane proteins, including FlhA and FlhB (reviewed in [30]). FliI is an ATPase forming a cytoplasmic complex with FliH and FliJ [31], [32], [33]. The six integral membrane proteins are postulated to form the export gate complex [34]. FliH-FliI-FliJ binds to export substrates and chaperone-substrate complexes [35], [36] and delivers them to the docking platform of the export gate made of the C-terminal cytoplasmic domains of FlhA and FlhB [37], [38]. ATP hydrolysis by FliI is proposed to release of the FliH-FliI-FliJ complex from the gate [39]. The export apparatus utilises the proton-motive force (PMF) across the cytoplasmic membrane as an energy source for unfolding and export of substrates [40], [41]. The membrane voltage component of the PMF is sufficient to support export in the wild-type export apparatus. However, the export gate complex intrinsically acts as a proton-protein antiporter that uses the two components of the PMF, the voltage and chemical potential gradients, for different steps of the export [41], [42].

We herein report the set-up of the screen and the findings that resulted from it.

## Methods

### Bacterial strains

A *Salmonella enterica* sv. Typhimurium strain, SJW1103 [43], was used as well as the derivatives from it listed in Table 1. They were stored as glycerol stocks at −80°C and propagated on Luria-Bertani (LB) agar plates or in LB broth with agitation, both containing the appropriate antibiotics, unless otherwise stated.

**Table 1:** List of strains and plasmids used in this work.

Strain	Description	Reference
SJW1103	*Salmonella* wild-type	[43]
MMHI001	Δ*fliH*-*fliI*	[66]
MMHI0117	Δ*fliH*-*fliI*, *flhB*_(P28T)_^*^; also known as Δ*fliH*-*fliI*, *flhB* ^*^	[40]
MMHIJ0117	Δ*fliH*-*fliI-fliJ*, *flhB*_(P28T)_^*^	[42]
DI15	Spontaneous drug insensitive and mildly hypermotile mutant	This work
	isolated from Δ*fliH*-*fliI*, *flhB*_(P28T)_^*^	
HM18	Spontaneous hypermotile mutant isolated from Δ*fliH*-*fliI*, *flhB*_(P28T)_^*^	This work
Δ*fliH*-*fliI*, *flhB*_(P28T)_^*^, Δ*atpB*	Deletion of the *atpB* gene in strain MMHI0117	This work
Δ*fliH*-*fliI*, *flhB*_(P28T)_^*^, Δ*fadB*	Deletion of the *fadB* gene in strain MMHI0117	This work
Δ*fliH*-*fliI*, *flhB*_(P28T)_^*^, *atpB*	Replacement of chromosomal *atpB* gene by the wild-type *atpB* gene	This work
Δ*fliH*-*fliI*, *flhB*_(P28T)_^*^, *atpBmut*	Replacement of chromosomal *atpB* gene by the allele	This work
	of the *atpB* gene found in HM18	

Plasmids	Description	Reference
pCP20	FLP encoding plasmid	[67]
pKD4	Template plasmid for gene disruption method	[49]
pUC19	High copy cloning plasmid	[68]
pWSK29	Low copy cloning plasmid, with T7 promoter removed	[69]
pIMA301	pUC19-*atpB*	This work
pIMA302	pWK29-*atpB*	This work
pIMA303	pWK29-*atpB mut*	This work
pMM108	pUC19 His-FLAG-*flhA*	[58]
pIMA306	pUC19 His-FLAG-flhA_A562P_	This work

### Provenance and handling of small molecule inhibitors

**Figure 1:**
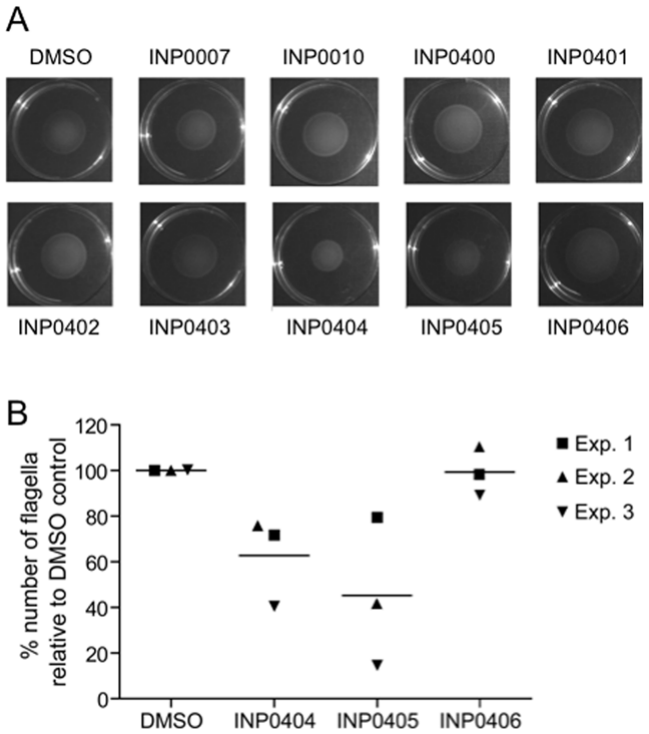
Effect of selected INP salicylidene acylhydrazides compounds on motility and flagella numbers in wild-type *Salmonella enterica* serovar Typhimurium SWJ1103. **A**) Motility of *Salmonella* in soft agar plates containing the compounds indicated, or the compound solvent DMSO as a control. An experiment representative of three repeats is shown. **B**) Electron microscopy analysis of flagella numbers on the surface of *Salmonella* exposed to INP0404, INP0405, INP0406 or DMSO. Three independent experiments were performed. For each sample and on each experiment 20–50 cells were scored and average number of flagella determined. In view of the variability of the numbers obtained from day to day, the data were normalised to the average number of flagella counted in the sample exposed to DMSO. These numbers were 4.1 (CI +/−1.5), 9.0 (CI +/−2.7) and 5.5 (CI +/−1.1) for experiments 1, 2 and 3, respectively. Lines represent the mean number for each condition. Using Poisson regression analysis of non-normalised data, INP0404 overall significantly reduced the number of flagella/cell (p<0.05). For INP0405, only experiments 2 and 3 showed significant reductions in the number of flagella/cell (p<0.001).

The compounds used here are named INP (by the company who produces them, which was formerly called Innate Pharmaceuticals and is now known as Creative Antibiotics at the University of Umeå, Sweden) followed by 4 numbers. In this study the main the drugs used were INP0404 (compound 20 in [15]), INP0405 (compound 9 in [15]) and INP0406 (used as a control, [22]). Their structures, and that of the other compounds used in Figure 1A, were published in Figure S1 of Veenendaal *et al.* (2009). The compounds were prepared according to literature protocols [44] and shipped as weighed powder in vials, where they can be stored in the dark at room temperature for many months. After addition of DMSO to generate 20 mM stock solutions, the vials were kept similarly and used within 1 month. As the drugs were used at either 50 µM or 150 µM (see below), the final DMSO concentration in all assays was either 32 or 96 mM.

### Motility assays and genetic screening

#### Motility assays

Motility plates were prepared using semisolid medium (1% Bacto Tryptone, 0.5%NaCl, 0.35% Bacto Agar). The medium was autoclaved and cooled, if required an inhibitor was added at 50 µM final concentration, and then it was rapidly poured into the plates before solidifying. Single colonies from the selected bacteria, freshly propagated onto LB plates as above the day before, were stabbed into the medium using sterile toothpicks. The plates were incubated at 30°C for 6–16 hours.

#### Genetic screening

A single colony of Δ*fliHI*/flhB* was inoculated into LB medium and grown for 8 hr at 30°C. Motility plates (10 cm in diameter) containing either 0.35% or 0.40% Bacto agar and either DMSO, INP0404 or INP0405 as outlined above were inoculated with 50 µl of bacterial culture using a Gilson pipette tip to gently open a central line in only the top half of the agar. Plates were incubated for 2 to 3 days at 30°C and regularly examined from day 2 for mutant emergence. Well-defined areas at the edges of the motility front where bacteria seemed to swim better than the majority of the inoculum were considered to contain candidate mutants. Toothpicks were used to collect bacteria from these areas and inoculate LB broth cultures, which were grown at 37°C overnight. Aliquots from these cultures were streaked onto LB agar plates and incubated at 30°C overnight to obtain single colonies. Three single colonies of each mutant were stabbed into motility plates containing DMSO, INP0404 or INP0405 using toothpicks. Each 10 cm plate accommodated 3 different candidate mutants and Δ*fliHI*/flhB* stabbed above and below the mutants in the same plate as a control. The plates were incubated at 30°C for 7 hr. Toothpicks were used to collect bacteria from 1 motility halo from each mutant which had retained the initial phenotypes observed (see Results) and these were used to inoculate LB broth cultures. Although mutants were initially isolated from plates containing either INP0404 or INP0405, they were subsequently rescreened on plates containing either drug in parallel. The re-isolation procedure was repeated at least 3 times to ensure isolate stability and homogeneity, before each candidate mutant was stored as a stock culture in glycerol. The overall screen was repeated several times with 10^9^ *Salmonella* being inoculated in total.

#### Quantitative motility assays

The quantity of cells applied with a toothpick from a plate colony is variable and this affects the reproducibility of the size of motility halos. To avoid this, colonies from the selected bacteria were cultured overnight in 5 ml LB medium at 37°C with agitation. An initial OD_600_ was taken in the next morning. Overnight cultures equivalent to 1 ml OD_600_ = 2.5 were pelleted by centrifugation at 2000 g for 10 min. Pellets were resuspended in 1 ml fresh LB medium and centrifuged as before. Finally, bacterial pellets were resuspended in 20 µl LB medium. 2 µl of each sample was stabbed into the motility plates. Both the control and the strain to be analysed were stabbed 2 or 3 times in alternation on the same plate using three plates per colony. The plates were incubated at 30°C for about 11 hours. Swimming halo radii were measured by hand.

#### Analysis of quantitative motility assays

“Drug insensitivity”: Averages of the radii were calculated per strain and the ratio of drug/DMSO control for a given strain (mutant or background) was calculated. At least 4 independent colonies were analysed per strain and drug. “Hypermotility”: Averages of the swimming halo radii were calculated on DMSO containing plates and hypermotility was assessed as the ratio of mutant/background strain for each individual plate (to normalise for differences between plates).

### Analysis of flagellar numbers

#### Morphology

Bacteria were cultured overnight in 5 ml LB medium at 37°C. Overnight cultures were diluted 50-fold in 10 ml LB broth. Drugs were added to a final concentration 50 µM. Control cultures were incubated with DMSO at the same concentration. Cultures were incubated at 37°C for 3–3.5 hours until they reached approximately OD_600_ = 1. Cultures equivalent to 2 ml OD_600_ = 1 were pelleted by centrifugation at 2000 g for 7 minutes at 4°C. Pellets were gently resuspended in 1 ml cold sterile-filtered PBS. Pellets were collected by centrifugation at 2000 g for 7 minutes at 4°C. Two µl of PBS were added to the pellets, which were resuspended by flicking. 5 µl of sample was applied directly on the glow-discharged, carbon-coated side of a 300-mesh Formvar coated copper electron microscopy grid and left for 1 minute at 25°C. The grid was put sample-side down on drop of double distilled sterile-filtered water for 1 minute and this step was repeated 4 times. Excess liquid was removed from the grid with filter paper and 5 µl of 0.25% (w/v) phosphotungstic acid pH 7 were added for 1 minute. Finally, the grid was dried with filter paper. Grids were observed in a FEI Tecnai12 Electron Microscope. Approximately 50 electron microscopy images were taken on a Tecnai12 transmission electron microscope (FEI) operating at 120 KeV. Micrographs were recorded at a magnification of 6 000 on an Eagle 4K×4K CCD camera (FEI) using the TIA software (FEI). Flagella numbers per individual cell were counted by hand.

*Statistical analysis*. As the flagella number/cell was skewed towards low numbers and often zero, we chose a Poisson regression to test for differences between experiments and between treatments.

### Growth measurements and analysis

#### Growth curves

Bacteria were grown overnight in 5 ml LB broth at 37°C. An initial OD_600_ was taken and then cultures were diluted 50-fold and incubated in 10 ml LB broth. In required, T3SS inhibitors were added to 150 µM. It was previously established that 50 µM of the drugs does not lead to any reduction in growth rate while a slight reduction is apparent at 100 µM ([14] and not shown). Cultures were shaken at 160 rpm at 37°C and the OD_600_ was taken every hour for 6 hours.

#### Growth rate determination

Simple bacterial growth curves can be described by sigmoidal functions using three parameters: the lag time λ, the maximal growth rate μ_m_ and the asymptote of the maximal cell density A. We have used a logistic growth model in the modification of Zwietering et al. 1990 [45]:
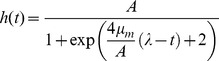
Curve fits were performed using gnuplot 4.2. All had fit errors below 15%. The maximal growth rates μ_m_ of three independent experiments were averaged and standard deviations were calculated.

### Genomic analysis

#### Genomic DNA isolation

Cells grown overnight (10 ml) were pellet by centrifugation, resuspended in 9 ml of PBS pH 7.4 and lysed with 1 ml of 20% SDS. Proteinase K (0.05 ml 20 mg/ml) was added and the mix incubated at 37°C until the cell suspension cleared due to lysis. RNase was added to eliminate RNA and the mix was incubated 10 min at 60°C. Proteins were extracted with phenol∶chloroform and chloroform∶isoamyl alcohol (24∶1). DNA was precipitated with 1/10 volume 3 M sodium acetate pH 5.2 and 2.5 volume ethanol and washed with 70% ethanol.

#### Whole genome sequencing

Whole-genome resequencing was performed using the Illumina GA2 sequencing system as described previously [46]. We generated 13,281,746 36-nucleotide reads from HM18; that is a total of 478,142,856 nucleotides, representing approximately 96-fold coverage of the 4.95-megabase genome. We generated 11,150,16136-nucleotide reads from DI15, representing approximately 81-fold coverage. To identify single-nucleotide variants, we aligned the Illumina sequence reads against the *S.* Typhimurium strain LT2 genome using the BWA alignment tool [47] and SAMtools [48]. Regions with less than 10× coverage or less that 95% consensus were considered ambiguous and excluded from analysis. One region of the LT2 genome, located between open reading frames STM2694–STM2772 and seemingly encoding a bacteriophage, was entirely missing from our data and therefore absent from the sequenced strain. Furthermore, any sequences present in ΔfliHI/flhB* that are absent from the LT2 reference would also have been omitted from the analysis since they would not have aligned to the reference genome. In total, of the 4,857,432 nt in the LT2 chromosome, 203,963 nt were ambiguous, leaving 4,653,469 nt of the LT2 genome as searchable for single nucleotide changes (SNCs).

### Chromosomal alterations of *atpB* and *fadB*

All alterations were generated using the λ Red system method [49].

#### Construction of atpB and fadB knockout strains

To construct the *atpB and fadB* mutant strains, the wild-type *atpB* or *fadB* genes were replaced by a kanamycin cassette in *Salmonella* ΔfliHI/flhB* as previously described [50]. To replace the wild-type *atpB* gene, a kanamycin resistance cassette was amplified using plasmid pKD4 as template and primers atpB_KO_for and atpB_KO_rev (Table 2) that contain 50 bp flanking regions homologous to the *atpB* gene. Same procedure was followed to replace the *fadB* gene using primers fadB_KO_for and fadB_KO_rev (Table 2). Mutants where the *atpB* or the *fadB* gene has been replaced by the kanamycin cassette were selected on plates with kanamycin and confirmed by sequencing.

**Table 2:** List of primers used in this study^a^.

Primer	Sequence
atpB_KO_for	ACTGGCGCCGGCTGTAATTAACAACAAAGGGTAAAAGGCATCATGGCTTCAGAAAATGTGTAGGCTGGAGCTGCTTC
atpB_KO_rev	ACAGTCTCCAGTTTGTTTCAGTTAAAACGTAGTAGTGTTGGTAAATTAATGCTCTTCGGACATATGAATATCCTCCTTAG
fadB_KO_for	ATCTGGTACGACCAGATCACTTTGTGGATTCAGGAGACTGACATGCTTTATAAAGGC GTGTAGGCTGGAGCTGCTTC
fadB_KO_rev	GCGAATAGCATCGACAATGACAACCTGTTCCATTGTGACTCCTTAAGCCGTTTTCAG**C**ATATGAATATCCTCCTTAG
atpB_XbaI_F	TGCTCTAGACAACAAAGGGTAAAAGGCATCATGG
atpB_EcoRI_rev	CCGGAATTCATTAATGCTCTTCGGACGCCATCGAC
atpB_XbaI_MUTF	TGCTCTAGACAACAAAGGGTAAAAGGCATCATTTCTTCAGAAAATATG
atpI_for	TTGGTGCTGGTGGTTCAGATACTGGCGCCGGCTGTAATTAACAACAAAGGGTAAAAGG GTGTAGGCTGGAGCTGCTTC
atpBwt_rev	CAGATGGTGTCCTATGTATTCCTGCGGCGTCATATTTTCTGAAGCCATGATGCCTTTTACCCTTTGTTGTTCATATGAATATCCTCCTTAG
atpBmutant_rev	CAGATGGTGTCCTATGTATTCCTGCGGCGTCATATTTTCTGAAGAAATGATGCCTTTTACCCTTTGTTGTTCATATGAATATCCTCCTTAG
flhA_XbaI_F2	CTAGTCTAGAGAGAAGAATACTGATGGCTAATCTGGTCGCGATGCTG
flhA_HindIII_rev	ACGCAAGCTTTTATTTTCCTCCAATGGTCGCCGTC
*flhA*_A562P_for	GGAAACGCTG**C**CGGAACATGCGCCGTTAC
*flhA*_A562P_rev	GTAACGGCGCATGTTCCGGCAGCGTTTCC

a The areas underlined correspond to restriction enzyme sites used and the base underlined and bold is the where the SNC is located that generates *flhA*_A562P_.

#### Chromosome replacement of the atpB gene

To replace the *atpB* gene by the *atpB* allele version found in HM18, a PCR fragment containing the *atpB* mutation and a kanamycin cassette was obtained using plasmid pKD4 as template and primers atpI_for and atpBmutant_rev. As a control, a wild-type version was obtained by the same procedure, using instead the primer atpwt_rev. The PCR fragments were inserted into *Salmonella* chromosome as above. All gene replacements were verified by sequencing. To eliminate the kanamycin cassette, plasmid pCP20 was used.

### Construction of *atpB* plasmids

To complement the *atpB* knockout strain with the *atpB* wild-type or mutant versions, the *atpB* gene was amplified by PCR using the ΔfliHI/flhB* strain as template and primers atpB_XbaI_F and atpB_EcoRI_rev for the wild-type version or atpB_XbaI_MUT and atpB_EcoRI_rev for the mutant version (Table 2). PCR fragments were digested with XbaI and EcoRI and cloned into plasmids pUC19 or pWSK29 digested with the same enzymes giving rise to plasmids pIMA301 and pIMA302 for the wild-type version (in pUC19 and pWSK29 respectively) and pIMA303 for the mutant version in pWSK29. All plasmids were verified by sequencing.

### Construction of mutant *flhA* plasmid

Plasmid pIMA306 (pUC19 His-FLAG-*flhA*_A562P_) was made by two-step PCR. The first step consisted of two PCR reactions, one carried out with primer flhA_XbaI_F2 and reverse primer flhA_A562P_rev containing the desired mutation and the other with flhA_A562P_for containing the mutation and *flhA*_HindIII_rev primers. The two PCR fragments were used as a template for the second PCR using primers flhA_XbaI_F2 and flhA_HindIII_rev. The PCR product was purified, digested with BsaMI and MluI and the 820 bp fragment was recloned into pUC19 His-FLAG-*flhA* digested with the same enzymes.

### Protein expression level measurements and secretion assays

Secretion assays for FliC and SipC were performed largely as previously described [51]. Briefly, bacteria were subcultured in LB broth, by 1∶100 dilution from overnight cultures, in presence of 50 µM of drugs or the equivalent amount of DMSO, for 6 to 7 hrs at 37°C with strong aeration. After measurement of culture optical density at 600 nm, the bacteria were pelleted by centrifugation at 2000 g for 10 min at 4°C. The supernatant was collected and filtered using a device with a pore size of 0.2 µm to remove any remaining bacteria. Bacterial pellets and supernatants were resuspended or diluted to equivalent cell densities and separated by SDS-PAGE. All supernatant samples were analysed by Silver stain prior to Western blotting to guard against occurrence of any bacterial lysis. The FlhA protein expression level measurements were performed similarly except that the bacteria were only grown until mid-exponential phase and that only the bacterial pellets were retained for analysis.

### Western blotting

This was performed as previously described [24] using an anti-FlhA rabbit polyclonal antiserum [52], an anti-FliC rabbit polyclonal antiserum [34] and an anti-SipC mouse monoclonal antibody (AC6; [53]). Signal quantification was performed by use of an Odyssey Imaging System (Li-Cor).

### Measurement of PMF components

Measurements of intracellular pH and membrane potential were performed as previously described [42], [54] in 10 mM potassium phosphate, 0.1 mM EDTA, and 10 mM sodium lactate after cells had been grown into mid-exponential phase in LB.

## Results

### INP0404 and INP0405 decrease the number of flagella observed per wild-type *Salmonella* Typhimurium cell

Negrea et al. (2007) [14] reported that, out of a selection of nine related salicylidene acylhydrazides used at 40 µM, only INP0404 and INP0405 significantly reduced the size of the swim zone of *S. enterica* serovar Typhimurium strain TT16729. We have found this to be true also in *S. enterica* serovar Typhimurium strain SJW1103, using the drugs at 50 µM (Figure 1A). In addition, they reported that at 40 µM these reduce levels of intracellular flagellin and, at 80 µM, flagellin surface presentation. Therefore, we were unsure what was leading to the drug-specific swimming defect observed. Since we had previously shown that some of these drugs prevent T3SS needle assembly, presumably by preventing needle protein secretion, in *Shigella flexneri* [24], we wanted to examine their effect on flagellar assembly in *Salmonella*. Figure 1B shows that both INP0404 and INP0405 reduce the number of flagella seen on the surface of SWJ1103 relative to control compound INP0406, although only INP0405 -which was also the stronger motility inhibitor in the work of Negrea *et al.* (2007)- did so to a statistically significant level. These data suggest that INP0404 and INP0405 act as they do in *Shigella*, i.e. by partially inhibiting the functions of both the flagellar and T3SS export apparatus. The reduced level of intracellular flagellin observed by Negrea et al (2007) could be explained by a well-known feedback loop that represses flagellin expression prior to hook completion [25].

### INP0404 and INP0405 inhibit the weak motility of *Salmonella* lacking genes encoding components of the cytoplasmic portion of the flagellar export apparatus

**Figure 2:**
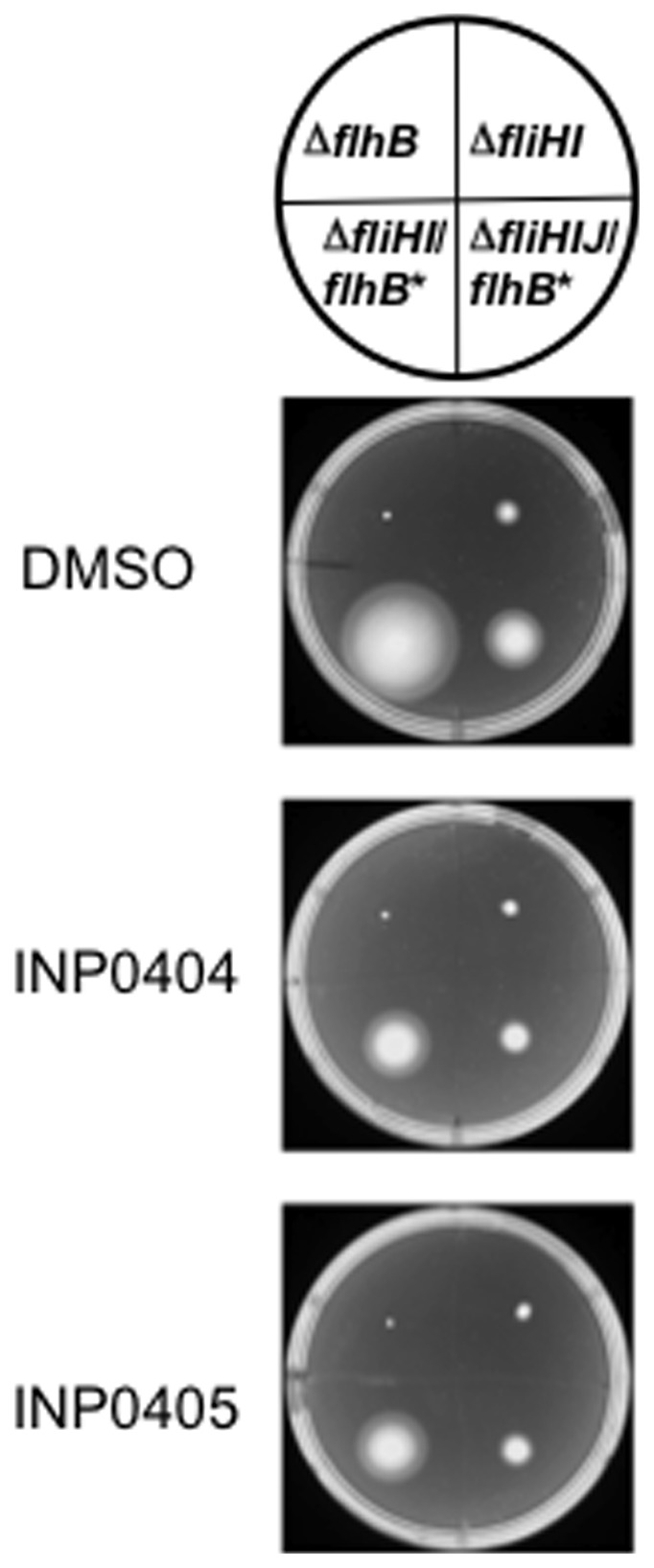
Effect of INP0404 and INP0405 on motility of weakly motile *Salmonella* Typhimurium strains lacking subunits of the cytoplasmic portion of the flagellar export apparatus. Motility assays were performed for the indicated strains in the position displayed on the circle above the plate photographs and using DMSO alone or the compounds indicated on the left. An experiment representative of two independent ones is shown. When the motility of ΔfliHI/flhB* and ΔfliHIJ/flhB* was expressed as a ratio of their motility in either drug relative to DMSO, the average values obtained were 0.69 and 0.73, respectively, for INP0404 and 0.65 and 0.61, respectively, for INP0405.

Recently, it was shown that *Salmonella* Δ*fliH-fliI* double null mutant strains lacking the flagellum-associated cytoplasmic ATPase FliI and its regulator FliH, have strongly reduced but not abolished motility. Accordingly, these strains also make very low numbers of flagella [40], [41]. This observation demonstrated that export of flagellar components could occur in the absence of the ATPase, which was previously thought to be the only export energiser, i.e. only in the presence of the membrane components of the export apparatus. These authors also showed that the residual export seen depended on the PMF across the cytoplasmic membrane [40], [41]. We wanted to use such strains to test whether any T3SS target of INP0404 and INP0405 was within the cytoplasmic or membrane components of T3SSs. Minamino & Namba isolated extragenic suppressor mutations in either of two flagellar inner membrane proteins, FlhA and FlhB, which enhanced the ability of the *fliH-fliI* double null mutant to form flagella. We used the most motile *fliH-fliI* bypass mutant, Δ*fliHI/flhB*_(P28T)_ -hereafter referred to as ΔfliHI/flhB*-, and a less motile mutant, ΔfliHIJ/flhB* [40], lacking a third soluble component of the export apparatus, FliJ [42], to test whether INP0404 and INP0405 could inhibit PMF-driven flagellar biogenesis. As shown in Figure 2, both drugs inhibit the motility of ΔfliHI/flhB*, that of ΔfliHIJ/flhB* and probably that of Δ*fliHI*. This indicates that FliH, FliI, and FliJ are unlikely to be targets of the drugs.

### Genetic screen for *Salmonella* mutants resistant to the motility-inhibiting effect of INP0404 and INP0405 in the ΔfliHI/flhB* background

**Figure 3:**
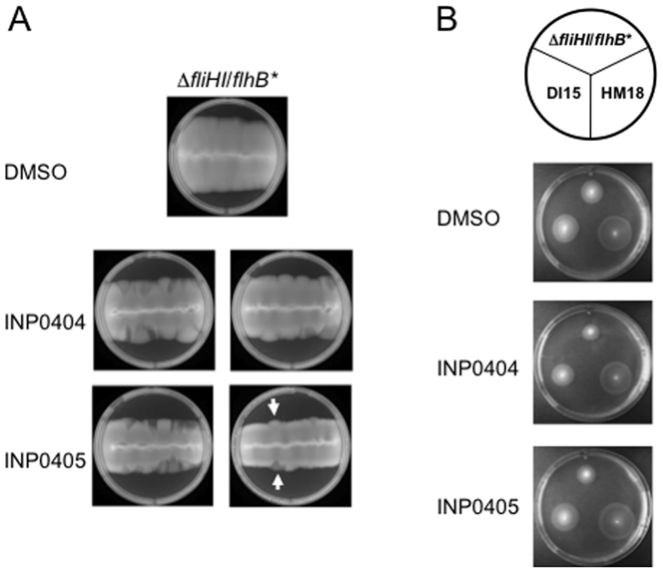
Isolation of drug resistant mutants from Δ*fliH*-*fliI*/flhB*. **A**) An example of the types of motility fronts obtained using the line method as described in the Materials & Methods. 0.4% agar plates were incubated for 3 days at 30°C. Note that leading edges of the fronts are smooth in the DMSO plate and ragged in the drug containing plates. In the latter, two types zones are seen at the edges, some translucent ones (*white arrow pointing upwards*) and some opaque (*white arrow point downwards*) ones, which are more similar to the rest of the front. Samples from both types of edge zones were taken for mutant isolation. **B**) Phenotypes of triply re-isolated mutants. Names of strains and mutants are indicated on the circle above the plates. Those that came from opaque (*left*) and translucent (*right*) areas of motility fronts were often DI and HM, respectively.

In the hope of identifying the molecular target(s) of INP0404 and INP405, we searched for mutants that were more motile than their parent strain in the presence of the drugs. This is done by picking colonies that emerge from the outer limit of the spreading motility fronts and hence swim better than their parent background (see Materials & Methods and Figure 3A). This was not possible to implement using wild-type *Salmonella*, which spreads to the edge of the plate even in the presence of the drugs during the few days necessary for mutants to emerge (not shown). Therefore, we decided to use ΔfliHI/flhB* as our background strain because it has slower motility due to its reduced number of flagella. As shown in Figure 3A, two types of colony morphologies were obtained that swam better than the background strain on drug containing plates. One was transparent and the other opaque, the latter being slightly rarer. Upon repeated re-isolation and phenotypic screening, as described in the Methods, it became apparent that the transparent clones swam better than the background strain in the presence and absence of the drugs (Figure 3B). They were hence termed hypermotile (HM) mutants. Opaque clones swam similarly to the background strain in the absence of the drugs but better than it in presence of the drugs and were hence termed drug-insensitive (DI). For both types of mutant, the mutation frequency relative to the initial bacterial cell input was calculated as approximately 10^−9^, when 8×10^−9^ to 4×10^−8^ is considered normal for rates of spontaneous mutation in *Salmonella* [55], indicating that the drugs were not themselves mutagenic.

**Figure 4:**
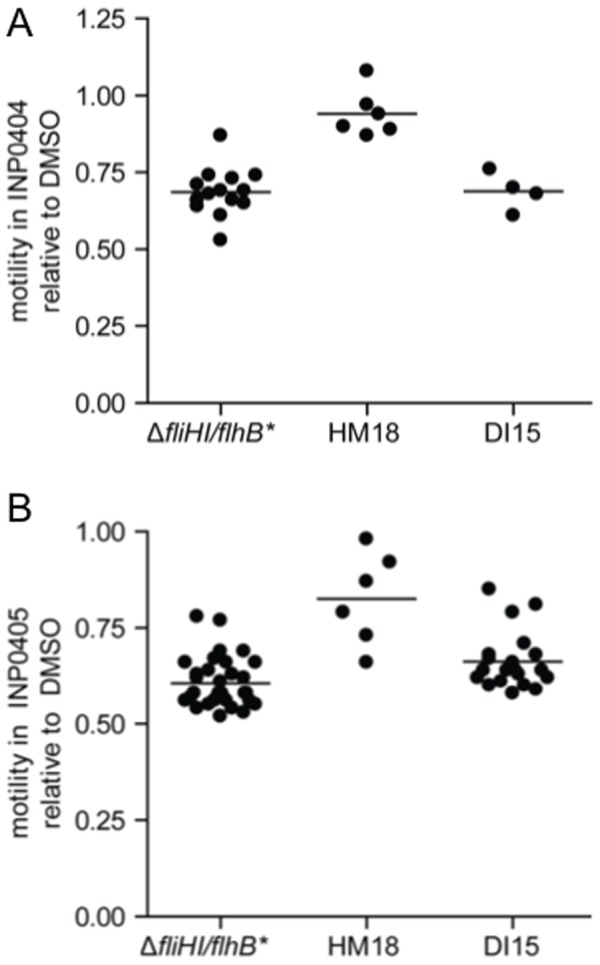
Quantitation of motility of HM18 and DI15 in INP0404 and INP0405 relative to ΔfliHI/flhB*. Quantitative motility assays were performed for the isolated mutants in the presence of **A**) INP0404 and **B**) INP0405. The motility of each strain is expressed as a ratio of its motility in either drug relative to DMSO. Dots represent individual colonies. Lines represent means. For INP0404, an Kruskal-Wallis test indicated a difference between groups (p = 0.0017) and a Dunn's post-test indicated that only HM18 (p<0.001) was significantly more motile than ΔfliHI/flhB*. For INP0405, a Kruskal-Wallis test indicated a difference between groups (p<0.0001) and a Dunn's post-test indicated that HM18 (p<0.001) and DI15 (p<0.05) were significantly more motile than ΔfliHI/flhB*.

**Figure 5:**
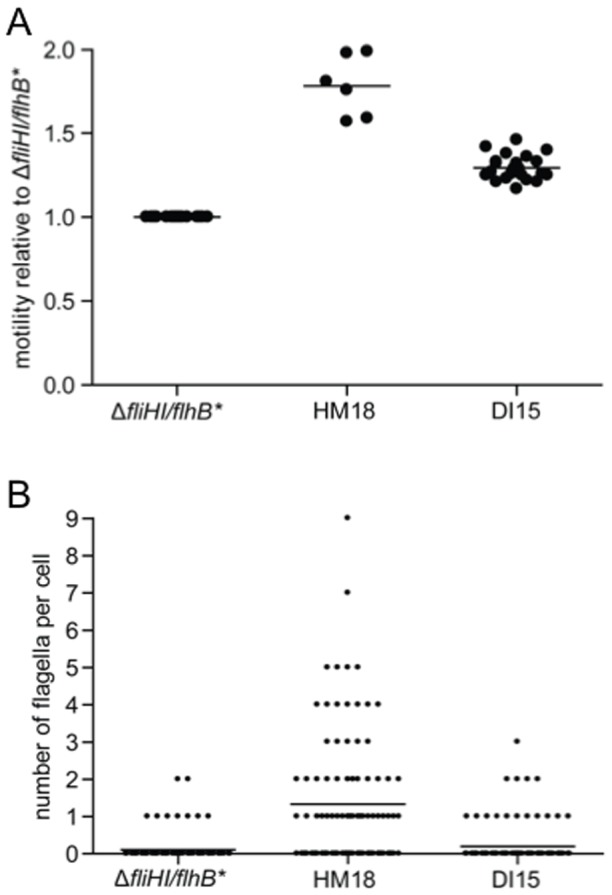
Hypermotility and flagellar numbers for isolated mutants. **A**) Quantitative motility assays of each mutant relative to ΔfliHI/flhB* in the presence of DMSO. Dots represent individual colonies. Lines represent means. Kruskal-Wallis test indicated a difference between groups (p = 0.0001) and a Dunn's post-test indicated that both mutant strains were significantly more motile than ΔfliHI/flhB* (p<0.001). **B**) Number of flagella for ΔfliHI/flhB*, HM15 and DI15. The display purposes, the data from two independent experiments were pooled. Lines represent means. Using Poisson regression analysis of unpooled data, HM18 had an overall significantly increased number of flagella/cell (p<0.001).

The two independent mutants, which had the strongest and most stable phenotype in their class, HM18 and DI15, were retained for further characterisation by quantification of their motility relative to the background strain in the presence of the drugs. HM18 appeared strongly resistant to the effects of INP0404 and INP0405 (Figure 4A & B). However, DI15 was only moderately resistant to INP0405 and not to INP0404. Measurement of the size of motility halos relative to that of the background strain in the presence of DMSO alone indicated that not only HM18 was hypermotile, but DI15 was also, albeit to a lesser degree (Figure 5A). Therefore, we found no mutant, which was strictly drug insensitive.

### Mutant HM18 is strongly hyperflagellated, has a growth defect and is resistant to the growth inhibitory effect of high INP0404 and INP0405 concentrations

We wanted to understand why the mutants we had isolated were hypermotile. Analysis of flagella numbers/cell showed that HM18 carried significantly more flagella than its parent strain ΔfliHI/flhB*, while DI15 did not (Figure 5B).

The translucent HM18 colonies on motility plates were suggestive of a growth defect. In addition, we had noticed that in the absence of drugs, HM18 cells were consistently ∼20% shorter and wider than ΔfliHI/flhB* cells during our morphological analysis of flagella numbers (not shown). Indeed, when its growth rate was assessed, it was found to be 3-fold lower than that of ΔfliHI/flhB* (Table 3, DMSO). However, DI15 grew normally (not shown).

**Table 3:** Growth rates of various mutants and strains under different conditions^a^.

Mutant/strain	DMSO	INP0404	DMSO/glucose^c^	INP0404/glucose
ΔfliHI/flhB*	1.064+/−0.101	0.495+/−0.042	1.703+/−0.101	1.196+/−0.112
HM18	0.373+/−0.019	0.290+/−0.019	1.011+/−0.145	0.824+/−0.073
ΔfliHI/flhB*^b^	1.137+/−0.130	0.577+/−0.106	NA^d^	NA
chr *atpB* WT				
ΔfliHI/flhB*	0.324+/−0.036	0.261+/−0.040	NA	NA
chr *atpB* mut				

a The growth rates of the strains were measured in the presence of the indicated amount of INP0404 or an equivalent volume of the drug solvent DMSO and the data processed as outlined in the Materials and Methods. Expressed as maximal growth rate (OD_600_/hr). Values given are averages of three independent experiments. Errors are standard deviations.

b Growth rates of ΔfliHI/flhB* where *atpB* has been replaced by *atpB* wild-type (chr *atpB* WT) or *atpB* carrying the mutation found in HM18 (chr *atpB* mut) within the chromosome.

c Where indicated, the strains were grown in the presence of 0.4% glucose (w/v).

d Not assessed.

We have shown previously that most salicylidene acylhydrazides drugs that inhibit T3SS secretion have slight growth inhibitory effects on *Shigella* when used above 100 µM [24]. Interestingly however, HM18 was insensitive to the growth-inhibitory effect of adding 150 µM INP0404 (Table 3) or INP0405 (not shown), unlike ΔfliHI/flhB*.

### Whole-genome sequencing of HM18 identifies mutations within *atpB* and *fadB* genes

To identify the mutations responsible for the phenotypes of both mutants, genomic DNA was extracted from strains ΔfliHI/flhB*, HM18 and DI15 and sequenced using Illumina's GA2 high-throughput sequencing technology.

When the sequence of mutant HM18 was compared to the one obtained for the ΔfliHI/flhB* parental strain, allowing non-ambiguous examination of 96% of the genome including the large plasmid (Table 4), three single-nucleotide changes (SNC) were identified within protein coding regions, specifically in the *atpB* (two adjacent SNCs) and *fadB* genes (1 SNC). *atpB* encodes the a-subunit of the F_O_F_1_-ATPsynthase and the mutation found in HM18 disrupts its translation start site. However, there is an in-frame start codon downstream that could be used as a new start site. Therefore, if translated, the mutant AtpB protein would lack its first 5 amino acids, which would affect its targeting to the inner membrane. In addition its concentration is very likely to be reduced due to less efficient translation initiation caused by distancing of the new start site from the Shine-Dalgarno sequence. *fadB* encodes the γ-subunit of fatty acid oxidation complex. In the HM18 strain, the protein is missing its last 147 amino as the mutation identified causes a premature stop codon.

**Table 4:** Summary of results of genomic analysis performed.

Chromosomal location of single nucleotide change (SNC)^a^	Present in strain	Nature of change	Position relative to nearby genes	Identification of gene affected	Likely consequence on protein product
**282402**	ΔfliHI/flhB*	C→A	Intergeneic region between pseudogene *cutF* and *proS* (prolyl-tRNA synthetase)	None	NA
**1248480** b	HM18	T→C	Intergeneic region between *dinI* (DNA damage inducible protein I and *pyrC* (dihydroorotase)	None	NA
**4080164**	HM18	C→A	NA	*atpB* (F0F1 ATP synthase subunit A)	Disruption of start codon, new in frame ATG 12 nt down stream, truncation of 5 amino acids of predicted periplasmic N-terminus of AtpB
**4080165**	HM18	C→A	NA	*atpB* (F0F1 ATP synthase subunit A)	Likely not translated due to SNC 4080164, new in frame ATG 11 nt down stream
**4189753**	HM18	G→A	NA	*fadA* (3-ketoacyl-CoA thiolase, also known as multifunctional fatty acid oxidation complex subunit α)	Stop codon introduced, last 147 C-terminal amino acids missing
**2008607**	DI15	C→G	NA	*flhA* (core inner membrane component of the flagellar export apparatus)	Single amino acid change introduced, A562P

a Defined by whole-genome Illumina sequencing and comparison to the *Salmonella* Typhimurium LT2 chromosome (RefSeq:NC_003197) and the 100-Kb plasmid pSLT (RefSeq:NC_003277). No differences were found between the plasmid of the LT2 reference and the two test strains.

b Note that this mutation (but no other) was also found in HM16 and DI13, which are not further described here, but not in ΔfliHI/flhB*. We thus assume it to be phenotypically silent.

### The *fadB* mutation is not responsible for the hypermotility of HM18

**Figure 6:**
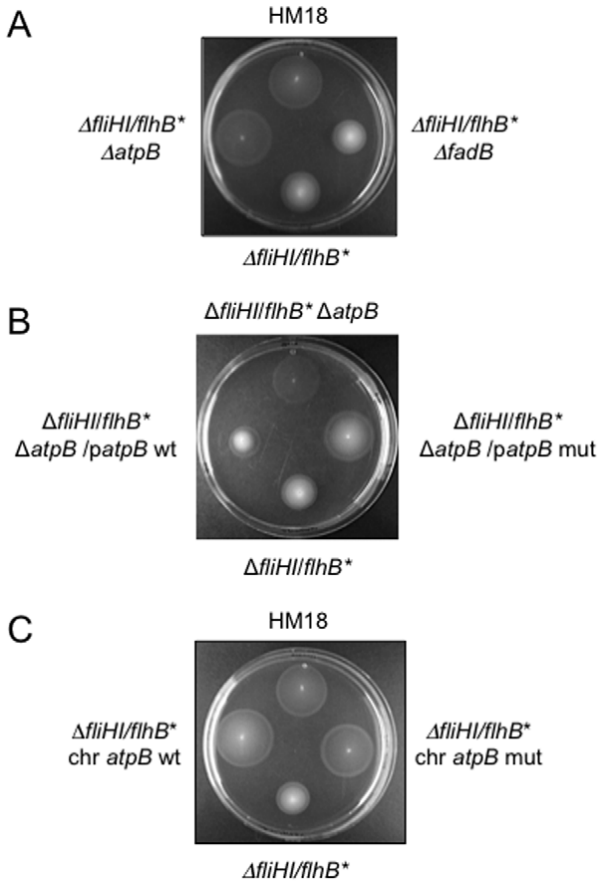
Analysis of the role of *atpB* and *fadB* in the hypermotility of HM18. **A**) Deletion of *atpB* but not *fadB* leads to hypermotility in the ΔfliHI/flhB* background. ΔfliHI/flhB*, ΔfliHI/flhB* Δ*atpB* and ΔfliHI/flhB* Δ*fadB* were stabbed into motility agar and incubated at 30°C for 16–18 hr. A representative experiment is shown. **B**) Extrachromosomal complementation of Δ*atpB* with wild-type but not mutant *atpB* restores weak motility to ΔfliHI/flhB*. ΔfliHI/flhB* Δ*atpB* and ΔfliHI/flhB* Δ*atpB* complemented with *atpB* wild-type (*atpB* WT) or *atpB* carrying the mutation found in HM18 (*atpB* mut) on a low copy plasmid were stabbed into motility agar along side ΔfliHI/flhB* and incubated at 30°C for 16–18 hr. A representative experiment is shown. Although Δ*fliHI*/flhB* Δ*atpB*/p*atpB* mut appears somewhat more opaque than the equivalently hypermotile Δ*fliHI*/flhB* Δ*atpB*, both it and Δ*fliHI*/flhB* Δ*atpB*/p*atpB* wt (which is not hypermotile) had similar growth defects in liquid culture. This suggests that their growth defects may be or appear somewhat different in motility medium. **C**) Chromosomal replacement of *atpB* by mutant and wild type *atpB* in ΔfliHI/flhB* both lead to hypermotility. Motility analysis for the same two strains in comparison to HM18 and ΔfliHI/flhB*. The plate was incubated at 30°C for 16–18 hr. A representative experiment is shown.

In order to investigate which mutation(s) is responsible for the hypermotile phenotype of HM18, *atpB* and *fadB* were independently deleted in ΔfliHI/flhB*. The motility of the new strains was compared with that of the ΔfliHI/flhB* and HM18 strains in soft agar plates (Figure 6A). The Δ*atpB* strain was hypermotile in comparison with ΔfliHI/flhB*. However no increase in motility was seen in the Δ*fadB* strain, indicating that alteration of *fadB* is not involved in HM18's hypermotility.

### The mutation in *atpB* causes HM18's hypermotility

In order to confirm the role of the mutation found in the *atpB* gene, ΔfliHI/flhB* Δ*atp*B was transformed with either the wild-type or the *atpB* allele found in HM18, hereafter termed the *atpB* mutant version of the gene, cloned in the low copy plasmid pWSK29. The motility phenotype of the different strains was analysed in soft agar plates (Figure 6B). Transformants carrying the *atpB* wild-type version behaved as the parental ΔfliHI/flhB* strain while transformants carrying the *atpB* mutant version were as hypermotile as Δ*atp*B. This indicates that the hypermotility phenotype in HM18 is caused by the *atpB* mutation. Since Δ*atpB* is also hypermotile, the *atpB* mutation found in HM18 may lead to loss-of-function by decreasing the AtpB concentration and/or the protein's functionality.

As we had previously noticed that HM18 has a growth defect compared to the parental strain (Table 3), growth curves were generated for these complemented strains. However, Δ*atpB* and Δ*atpB* carrying pWSK29 with either wild-type or mutant *atpB* all showed a similarly strong defect in growth compared with the parental strain (not shown). In addition, when the ΔfliHI/flhB* Δ*atpB* was complemented with wild-type *atpB* cloned in a high copy number vector, the growth defect was even greater (not shown). Taken together, these results suggest that either an increase or a decrease in AtpB concentration can cause a growth defect.

### Chromosomal replacement of *atpB* by mutant but not wild-type *atpB* replicates HM18's growth defect

As expression from the chromosome would ensure that *atpB* is expressed at similar levels as in the background and HM18 strains, we replaced the *atpB* gene with the mutant version in the parental ΔfliHI/flhB* chromosome using the λ Red system [49]. When this method is used a “scar” (an insertion of few nucleotides) is left in the chromosome, therefore a wild-type *atpB* version was inserted following the same procedure as a control. When growth curves were generated for the new strains we observed that the *atpB* wild-type replacement had a similar growth rate compared with the parental strain and the *atpB* mutant replacement was similar to the HM18 mutant, including in terms of insensitivity to the inhibition of growth induced by 150 µM INP0404 (Table 3). However, when we analysed the motility phenotype of these new strains, we found both to be hypermotile (Figure 6C).

We conclude that changes in *atpB* expression levels and/or its coding sequence cause both hypermotility and growth defects, although which of the two phenotypes is displayed depends on the level at which the *atpB* alleles are expressed.

### Glucose suppresses the growth defect induced by high drug concentrations in ΔfliHI/flhB*

It is well known that defects in the *atp* operon of Gram negative bacteria prevent oxidative phosphorylation. In order to survive, bacteria then increase substrate level ATP synthesis via upregulation of glycolysis [56], [57]. In LB medium, as used so far, bacteria are predominantly using amino acids as carbon and energy sources, which are a poor source of ATP via glycolysis when oxidative phosphorylation is blocked by mutation. To test whether at 150 µM INP0404 is directly targeting the oxidative phosphorylation pathway and so causing growth to slow in LB, we sought to stimulate ATP production via substrate level phosphorylation during glycolysis by adding glucose to the medium. As shown in Table 3, addition of glucose stimulates growth of ΔfliHI/flhB* and HM18. Glucose also partially suppresses the growth defect induced by 150 µM INP0404 in ΔfliHI/flhB* (INP0404/DMSO ratio is 0.47 versus INP0404+glucose/DMSO+glucose ratio of 0.70) but leaves HM18 unaffected (ratios with and without glucose of ∼0.75). This indicates that the resistance to the drug-induced growth defect in HM18 is not due to its reduced growth rate but related instead to the physiological pathway altered within it. Since we have shown that in HM18 the relevant altered pathway is oxidative phosphorylation, we conclude that the drugs may be targeting this biochemical pathway.

### Molecular characterisation of DI15

Whole genome sequencing of DI15 identified a single SNC, when compared to that of ΔfliHI/flhB* within *flhA* of the flagellar inner membrane export apparatus that leads to FlhA_A562P_. Since ∼96% of the genome of ΔfliHI/flhB* was searchable for SNCs and no other was found, it is very likely to be solely responsible for the phenotype seen in this background. However, when we tried to complement Δ*flhA* ΔfliHI/flhB* with *flhA*_A562P_ versus *flhA*, using the pMM108 His-FLAG-FlhA complementation plasmid [58] and inserting the *flhA*_A562P_ allele to generate a second version of this plasmid, we were unable to assess the DI phenotype because restoration of motility with WT *flhA* was too poor even upon extended incubation times (data not shown). We next examined the effect of FlhA_A562P_ in an otherwise wild-type background. For this we used a previously generated Δ*flhA* strain [59]. Although variable, complementation of this strain with *flhA* was more successful. Yet, relative to *flhA*, the *flhA*_A562P_ allele alone did not lead to insensitivity to INP0405 or to the slight hypermotility observed in DI15 (data not shown).

### Influence of HM18 and DI15 backgrounds on T3SS function

**Figure 7:**
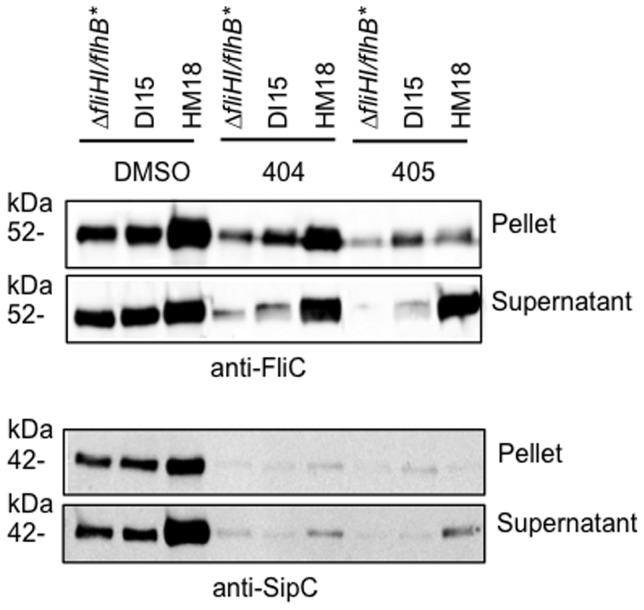
Influence of the DI15 and HM18 backgrounds on the function of flagellar and virulence-related T3SSs. Strain ΔfliHI/flhB*, DI15 and HM18 were grown to late exponential phase in the presence of DMSO, or 50 µM INP0404 or INP0405 and normalised for OD_600_. Cultures were separated into cell *pellet* and culture *supernatant* by centrifugation, followed by SDS-PAGE and Western blotting with anti-FliC (flagellin, *top*) and anti-SipC (SPI1 T3SS effector, *bottom*) antibodies. A representative experiment is shown.

We wanted to understand how the mutations we had uncovered affected not only flagellar but virulence-related T3SSs. In *S.* Typhimurium, two 2 virulence T3SSs exist, SPI1 and SPI2 [14]. SP1 is required for invasion of eukaryotic host cells and SPI2 for replication inside them, within a membrane-bound vacuole. SPI1 is expressed in LB but SPI2 expression requires growth in a medium low in nutrients and magnesium at pH 5 [60]. As expected, HM18 grew very poorly in MgM-MES (not shown). Therefore, we could not establish the effect of our mutant backgrounds on the SPI2 secretion system. However, we were able to examine expression and secretion of FliC by the flagellar T3SS and of SipC, by the SPI1 secretion system (Figure 7). FliC and SipC expression and secretion are increased approximately 2-fold in HM18, relative to either DI15 or ΔfliHI/flhB* in the presence of DMSO. However, their expression is strongly reduced (2 to 7-fold) during growth in the presence of either drug, as previously reported [14], in all three strains examined here. Despite this, the secretion of FliC is almost unaffected by addition of either drug in the HM18 background, while it is also not fully reduced to ΔfliHI/flhB* levels in DI15 (Figure 7, top panel). This indicates that secretion of FliC is oblivious to drug addition in the HM18 background, while it is less sensitive to drug addition in the DI15 background. Expression of SipC is almost abolished in the presence of either drug (Figure 7, bottom panel), as previously reported [14], in all strains tested. However, SipC secretion is still ∼2-fold higher in HM18 than in ΔfliHI/flhB* or DI15. Taken together, these data indicate that the effect of the *flhA* mutation in DI15 is autonomous to flagellum assembly while that of the alteration of *atpB* in HM18 is not.

## Discussion

It was proposed that drugs inhibiting virulence-associated instead of “house-keeping” functions in bacteria would lead to reduced resistance emergence [61]. Evidently, the frequency of mutant emergence found here corresponds to that of spontaneous mutation in *Salmonella*. Moreover, in the host, where virulence-like T3SS are essential to survival and replication, and flagellar T3SS are important for host colonisation by commensals and pathogens alike, presence of T3SS inhibitors may generate a selective force for resistance emergence. However, there should be less pressure for general drug handling mechanisms, which are easily mobilisable and hence transferable, to evolve since their targets are non-essential. In addition, resistance to virulence inhibitors should not pre-exist in the environment, with novel target site mutations being especially poorly mobilisable since the functions targeted are not as conserved as those targeted by antibiotics.

Study of the development of antibiotic resistance showed that this occurs by four main mechanisms: 1) enhanced drug export, reduced entry or degradation, 2) up-regulation of expression of the drug target and 3) mutation of the drug target or 4) acquisition of a modified target [1]. When screening for resistance to antibiotics, the first 3 classes of mutants are often identified. HM18 did not reveal mutations in genes encoding the putative T3SS target(s) of the drugs that our work in *Shigella* [24] and *Salmonella* (above) had led us to expect. We discuss first why we think HM18 is an unusual representative of a class 2 mutant. On the other hand DI15 does carry a mutation within a flagellar export apparatus but we discuss further below why we do not think it represents a class 3 mutant and may therefore be a weaker representative of a class 2 mutant.

### What does the study of HM18 indicate about flagellar protein secretion and the mechanism(s) of action of the drugs?

#### Hyperflagellation

Our complementation data show that the alteration in *atpB* within HM18 is responsible for both the hyperflagellation, causing an increase in motility, and for its growth defect. Alterations of the *atpB* operon are known to cause growth defects [56]. Indeed, when we deleted *atpB* in the wild-type background SJW1103, we saw the same growth defect, but we did not see an increase in motility (not shown). Therefore, how might an alteration in *atpB* in the Δ*fliHI*/flhB* background cause hyperflagellation?

Under aerobic growth conditions, as used in our work, the PMF generated by the respiratory electron transport chain across the cytoplasmic membrane is used by the F_O_F_1_-ATP synthase to generate ATP. Loss-of-function in the *atp* operon would lead to a decrease in intracellular ATP levels and an increase in free protons in the periplasm, to a point where the electron transport chain stops and NADH levels build up, blocking the remaining source of ATP, i.e substrate-level phosphorylation via glycolysis. However, this does not occur because bacteria have evolved compensatory mechanisms to deal with this scenario, including upregulation of glycolysis to provide ATP by substrate level phosphorylation and upregulation of certain components of the electron transport chain to allow faster re-oxidation of NADH whilst avoiding an increase in the PMF [57].

**Figure 8:**
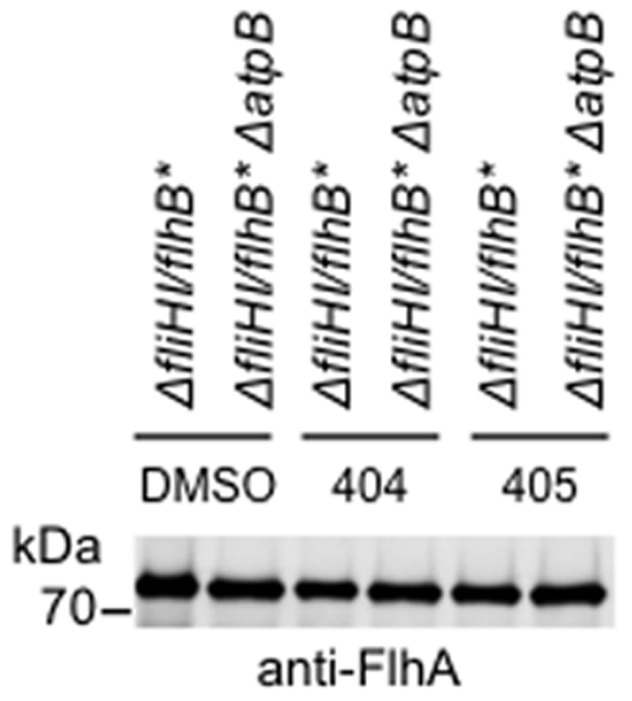
Measurement of FlhA expression in ΔfliHI/flhB* under different conditions. Whole cell extracts of ΔfliHI/flhB* carrying or not the chromosomal deletion in *atpB*, grown to mid-exponential phase in the presence of DMSO, or 50 µM INP0404 or INP0405 and normalised for OD_600_, were separated by SDS-PAGE and Western blotted with an anti-FlhA antiserum. No difference in FlhA level was observed under any of the conditions tested.

In Δ*fliHI*/flhB*, flagellar protein export is entirely driven by the PMF [40]. Hence, in this background, an increase in the PMF seen in the absence of sufficient or functional F_O_F_1_-ATP synthase may be dissipated by a proton-conducting channel within the flagellar export apparatus. This is consistent with similarly increased motility in a strain lacking a component of the flagellar C-ring (which supports the function of cytoplasmic export apparatus) and carrying a deletion of *atpA* [62]. This process would stimulate export of flagellar proteins (as seen in Figure 7), hence stimulating flagellation, and at the same time, allow re-oxidation of NADH by the electron transport chain, facilitating ATP synthesis by substrate-level phosphorylation in glycolysis. Indeed, Figure 8 shows that the hypermotility seen in ΔfliHI/flhB* Δ*atpB* is probably not due to an increased number of basal bodies, as evidenced by the similar levels of FlhA detected by Western blotting in this strain versus ΔfliHI/flhB* Indeed, the number of basal bodies formed per bacterium is known to be regulated upstream of filament completion [25]. Hence, the hypermotility of this strain -and HM18- is most likely due to an increase in the number of basal bodies able to polymerise a flagellum.

However, we measured the PMF components in SJW1103, Δ*fliHI*/flhB*, SJW1103 Δ*atpB* and Δ*fliHI*/flhB* Δ*atpB* and found no difference in the overall PMF in any of these strains (not shown). Therefore, if the export apparatus in the flagellar T3SS does serve to dissipate some of the increased PMF in Δ*fliHI*/flhB* when *atpB* is deleted, the differences produced in the PMF components are too small or too local to be detectable in SJW1103 under our experimental conditions Δ*atpB* [57].

### Apparent resistance to drug-induced motility defect

During our experiments with *atpB-*modified strains, we noticed a correlation of hypermotility with drug resistance and not with growth defects. First, chromosomal replacements of *atpB* by mutant and wild-type forms in ΔfliHI/flhB* are both hypermotile and also seemingly resistant to INP0404 and INP0405 (not shown), but only the mutant replacement leads to a growth defect. Second, plasmid complementation of Δ*atpB* by either a wild-type or mutated version of the gene leads to a growth defect. However, only Δ*fliH*I/flhB* Δ*atpB*/p*atpB* mut is hypermotile and drug resistant, whereas Δ*fliH*I/flhB* Δ*atpB*/p*atpB* wt is neither. Therefore, apparent drug resistance is linked to hypermotility in these strains. We propose that HM18 seems drug resistant because it is hyperflagellated. Since the intracellular amount of a flagellar basal component is unaltered (Figure 8), this is unlikely to represent a typical class 2, “target upregulation”, mutant. Instead, it is rather a case of “function upregulation”. As it secretes more flagellin (Figure 7), HM18 probably produces more flagella than ΔfliHI/flhB* in absence of any drug. Addition of either drug reduces the number of flagella HM18 produces (not shown) and its motility, indicating that the mutant is still sensitive to the effects of the drugs on flagellation. But, the drugs do not affect HM18's ability to express and secrete flagellin as much as they do for ΔfliHI/flhB*. In addition, motility probably does not correlate linearly with number of flagella, of which ΔfliHI/flhB* has fewer to start with, adding to appearance of drug resistance by HM18.

### Resistance to drug-induced growth defect

**Figure 9:**
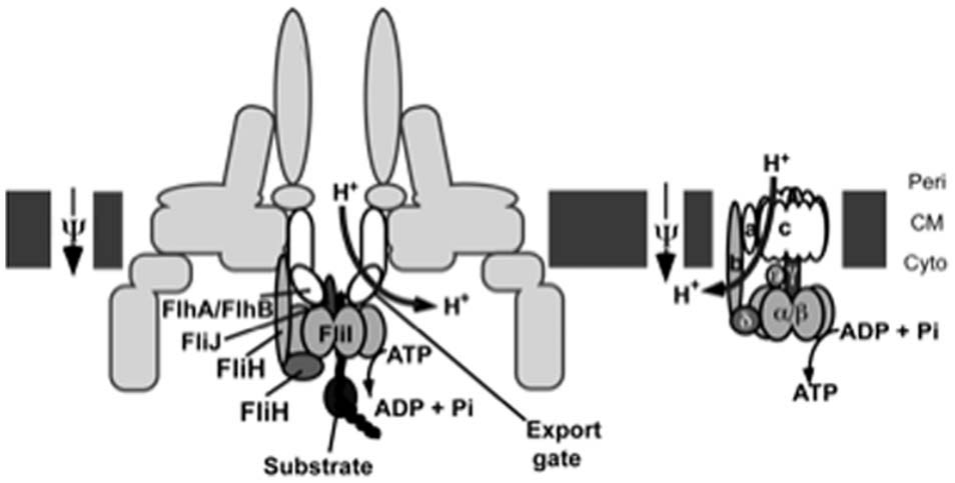
Comparative schematic representation of the bacterial flagellar export apparatus and F_O_/F_1_ ATPase. The bacterial cytoplasmic membrane (*CM*) is shown in dark gray blocks. *Peri* stands for periplasmic face and *Cyto* for cytoplasmic face. The names components that are functionally and/or structurally related between the two complexes (as described in the Discussion) are outlined in black and share similar colours and/or shapes.

HM18 displays a growth defect. In addition, it is less sensitive to growth-inhibitory effect of high concentrations of the drugs than ΔfliHI/flhB*. This suggests that at these concentrations at least, the drugs are detectably influencing the oxidative phosphorylation pathway, which is defective in HM18. As HM18 displays near complete resistance to the drug-induced growth defect, it may be that at 150 µM the drugs are targeting AtpB directly. Many small molecules inhibitors of mammalian and/or bacterial ATP synthase are known [63], [64]. Alternatively, in view of the results of Wang et al. (2011), the physical targets of the drugs in this pathway may be elsewhere. Indeed, these authors identified two proteins, WrbA and Tpx, which are involved in oxidative metabolism, as directly interacting with salicylidene acylhydrazides [29]. This suggests that a primary target of these drugs may instead be within the electron transport chain. Their effect on the electron transport chain may even have contributed positively to selection of the *atpB* mutation in our screen. Indeed our *atpB* mutation would be expected to uncouple ATP synthesis from a membrane-functional electron transport chain due to its stimulation of substrate-level phosphorylation [65] and this would be beneficial to bacteria growing under the pressure of a drug that inhibits the electron transport chain (Figure 9).

### Link between the PMF, flagellation, motility and growth

If the drugs really were inhibitors of the electron transport chain, they should lead to a reduction in proton concentration in the periplasm. This would explain why they reduce flagellation in and hence ultimately motility of HM18 in soft agar. This is also consistent with the fact that we and others found that at 50 µM, their inhibitory effect on T3SSs requires a pre-treatment of at least 30 min, i.e. ∼1 bacterial generation time, but is rapidly reversible [15], [24]. However, at 50 µM the drugs do not detectably reduce swimming speeds of *S.* Typhimurium in liquid culture (not shown), suggesting that the proton-driven rotary flagellar motor is unaffected by any reduction in periplasmic protons induced at this drug concentration. Finally, bacterial growth is only affected beyond 100–150 µM of the drugs. Overall, this indicates that probably flagellar and virulence T3SSs are sensitive to smaller decreases in the PMF than flagellar rotation and growth are.

### The DI15 mutation probably does not identify a target of INP0405 within the flagellar T3SS apparatus

Only one SNC was found in DI15's genome, which leads to the A562P mutation within FlhA, a polytopic inner membrane protein of the flagellar export apparatus. This suggested that its weak insensitivity to INP0405 is specific to this SNC. However, for technical reasons, we could not test this directly via complementation of Δ*flhA* ΔfliHI/flhB* and when we examined the effect of *flhA*_A562P_ in a Δ*flhA* background, we found that it did not lead to either the mild INP0405 resistance or the mild hypermotility observed in DI15. This suggests that the *flhA*_A562P_ does not mediate resistance directly. Instead, it may do so indirectly, perhaps via enhancement of the effect of the flhB* mutation in the Δ*fliHI* background. Indeed, several flhA* alleles were previously found in the Δ*fliHI* background [40]. If the drugs did lead to a decrease in the number of protons in the periplasm, any enhancement of flagellation by *flhA*_A562P_ in ΔfliHI/flhB* would lead to apparent resistance (Figure 9). Within this framework, DI15 would be insensitive to INP0405 and not INP0404 (Figure 4) because, of the two, INP0405 is the strongest inhibitor of motility and flagellation (Figure 1) and therefore, probably also of the electron transport chain. Hence INP0405 would create the greatest flagellation and hence motility differential between DI15 and ΔfliHI/flhB*. This would be in agreement with the findings of Tree et al. (2009; [26]) who identified a strain of *E. coli* O157, ZAP-430, that showed no inhibition of T3SS secretion by four different salicylidene acylhydrazides. Sequencing of its genome and comparison against an *E. coli* strain that remained sensitive to the effects of the drugs on T3SS secretion identified numerous SNCs but none in T3SS operons, suggesting a lack of direct T3SS targets for these drugs.

### INP0404 and INP0405 may affect virulence-related T3SSs in the same indirect manner as they affect flagellar biogenesis

Finally, we showed that SPI1 function is upregulated in HM18 but not in DI15 and that in HM18 it is mildly resistant to drug addition. The mildness of this effect is probably the consequence of two facts: 1) the drugs severely decrease SPI1 gene expression [14] and 2) in ΔfliHI/flhB* and its derivatives, the SPI1 secretion system, unlike the flagellar one, still has a functional export ATPase. When this protein complex is present, it affects how the PMF is used in T3SS-mediated export, making its role less apparent [40], [42]. Nevertheless, these findings support the notion that flagellar and virulence-related T3SS are similarly affected in HM18, but not in DI15. The *flhA* mutation in DI15 would be expected to be autonomous to flagellar function because it lies in a gene encoding a flagellar component. However, that the *atpB* mutation in HM18 also affects SPI function alone and in the presence of the drugs suggests that these may be affecting virulence T3SSs in the same manner as they do flagellar ones.

All together therefore, our findings suggest that salicylidene acylhydrazides are not direct “virulence blockers”. Instead, they affect virulence indirectly via a generalised, if low level at the concentrations initially used, disruption of basic bacterial physiology. In the long-term, such virulence blocking small molecules may be easier to identify using rational design, or high-throughput screens on defined molecular targets rather than on whole cell assays.
